# How to recognize if a cell dies from pyroptosis?

**DOI:** 10.1007/s11033-026-12347-z

**Published:** 2026-07-15

**Authors:** Michał Gebuza, Weronika Bihl, Kinga Brawańska – Maśluch, Paulina Tomecka, Marek Kulbacki, Julita Kulbacka

**Affiliations:** 1 Department and Clinic of Ophthalmology, Medical University Hospital, Borowska 213, Wrocław, 50-556 Poland; 2 Lower Silesian Centre for Oncology, Pulmonology and Hematology, Plac Ludwika Hirszfelda 12, Wrocław, 53-413 Poland; 3 Department of Physiology, Faculty of Physiology and Pathophysiology, Medical University of Wrocław, Wrocław, Poland; 4 Medical University Hospital, Borowska 213, Wrocław, 50-556 Poland; 5 Polish-Japanese Academy of Information Technology, Koszykowa 86, Warsaw, 02- 008 Poland; 6 Department of Molecular and Cellular Biology, Faculty of Pharmacy, Wroclaw Medical University, Borowska 211A, Wroclaw, 50-556 Poland; 7 Department of Immunology and Bioelectrochemistry, State Research Institute Centre for Innovative Medicine, Santariškiu˛ g. 5, Vilnius, LT-08406 Lithuania

**Keywords:** Cell death, Programmed cell death, Pyroptosis, Canonical pathway, Noncanonical pathway, Cancer cells

## Abstract

Pyroptosis is a distinct form of programmed cell death and plays a crucial role in immune processes by eliminating pathogens. It is mediated by the inflammasome pathway and is characterized by the release of proinflammatory cytokines. Distinguishing pyroptosis from other forms of programmed cell death, such as apoptosis and necroptosis, is essential for understanding its biological function in various physiological and pathological processes. In cancer development, pyroptosis has a dual role. Depending on the context and proper stimulation, it can either promote tumor growth and progression or lead to tumor destruction and suppression. The detection of pyroptosis-related molecules can serve as prognostic indicators and potential therapeutic targets across various treatment modalities. This review presents the methods and key factors involved in deciphering the occurrence of pyroptosis in cells, offering valuable insights into its role in diverse biological processes and clinical applications.

**Graphical abstract:**

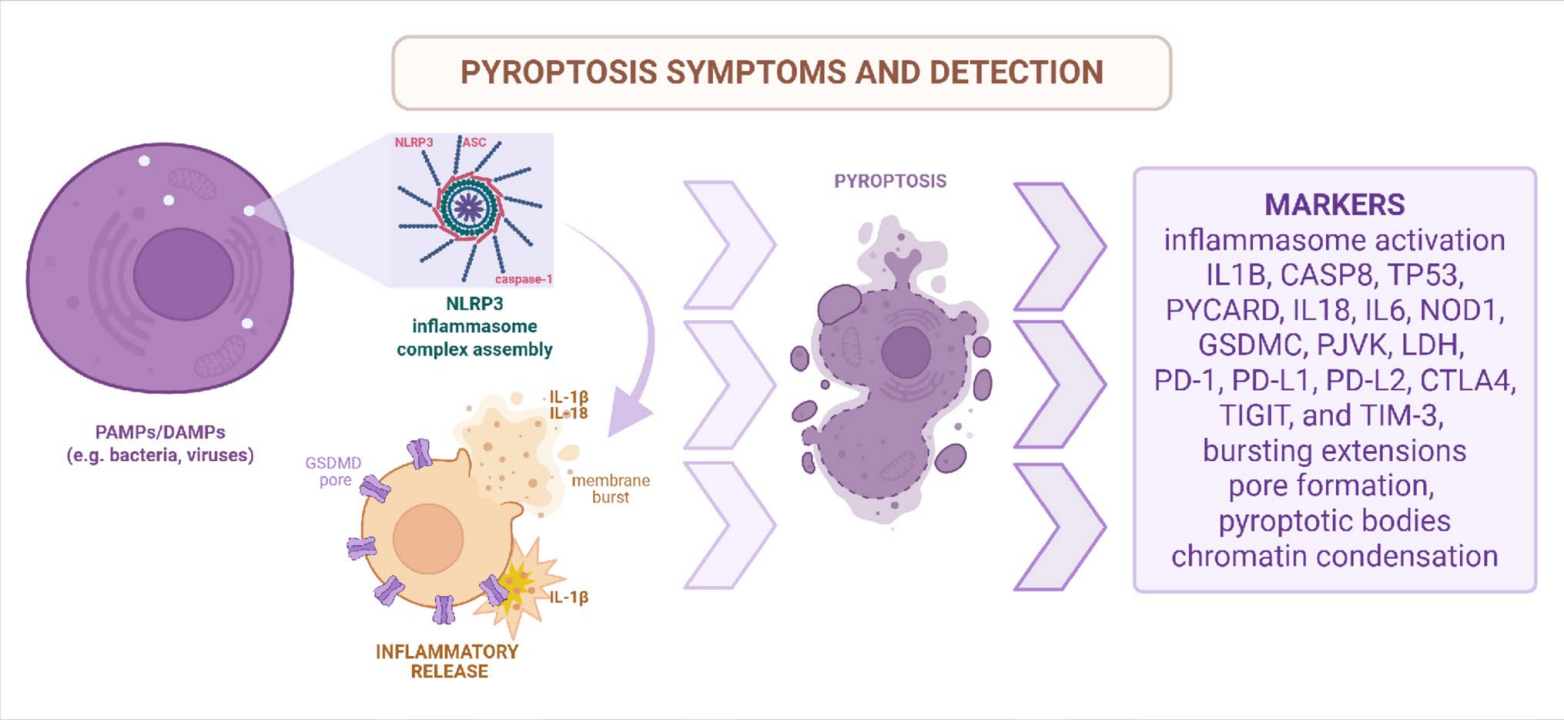

## Introduction

The first mention of the pyroptosis process was in 1986, when it was described as an efflux of cell contents caused by the lethal toxin anthrax [1]. In 1992, Zychlinsky *et al*. described for the first time Pyroptosis [2]. However, the name Pyroptosis started to be proposed as a new form of programmed cell death (PCD) in 2001 [3]. Pyroptosis - the prefix pyro means “fire or fever” from Greek, and the suffix ptosis means “falling or to fall down”. Among other patterns of PCD, pyroptosis is a type of pro-inflammatory programmed cell death [3]. Various programs can cause cell death, including autophagy, oncosis, and programmed cell death via caspase-1 (referred to as pyroptosis). At the molecular level, pyroptosis is defined by activation of the inflammatory caspases - caspase-1 in the canonical inflammasome pathway (NLRP3, AIM2, NLRC4) and caspase-4/−5/−11 in the non-canonical pathway - which cleave gasdermin D (GSDMD); the unconventional N-terminal fragment forms oligomers that create plasma membrane pores, facilitating the release of IL-1β and IL-18 and inducing lytic cell death. This molecular signature distinguishes pyroptosis from apoptosis, which is executed by caspase-3/−7 (downstream of caspase-8/−9), proceeds without membrane rupture, and produces apoptotic bodies in an immunologically silent manner, and from necroptosis, which is caspase-independent and instead relies on RIPK1–RIPK3-mediated phosphorylation and oligomerization of MLKL to permeabilize the plasma membrane [4, 5]. Unlike apoptosis, a controlled, non-inflammatory process, pyroptosis is a form of PCD that occurs in response to certain cellular signals, such as infection or inflammation. Pyroptosis is characterized by the release of pro-inflammatory cytokines and the formation of pores in the plasma membrane, causing cellular swelling and leading to cell lysis and intracellular contents [4, 6]. Currently, many researchers are focusing on the role of pyroptosis in identifying new therapeutic targets. One of the main areas of interest in this process is oncology. According to the World Health Organization (WHO), cancer deaths will increase by 80% by 2030 [4, 6]. Cancer ranks as the primary cause of death in developing countries, whereas in developed countries, it is the second most common cause of death [7]. This process now has many possible applications for treating various diseases, including melanoma, breast cancer, lung and cervical cancers, colon cancer, infectious diseases, and some neurodegenerative disorders [8, 9]. Pyroptosis is also an important part of the immune response to pathogens, as it helps to eliminate infected cells and stimulate an immune response. However, improper regulation of pyroptosis can also contribute to the development of inflammatory diseases, such as sepsis, autoimmune disorders, and neurodegenerative diseases [10].

## Triggering of pyroptosis

Pyroptosis is a type of PCD that occurs when the balance between the cell’s internal and external environments is disrupted, often due to innate immune responses to pathogens. It assists cells in their functioning by eliminating intracellular replication niches. Distinct morphological changes characterize this process. It is triggered by inflammatory signals, such as pathogen-associated molecular patterns (PAMPs) or damage-associated molecular patterns (DAMPs) released by damaged or dying cells. In the process of pyroptosis, further inflammasomes are created, which are large intracellular protein complexes that cleave pro-caspase-1 into the active form, which in turn cleaves the pore-forming protein gasdermin D (GSDMD) to produce N-terminal fragments. Then, they form membrane pores in the cell membrane, leading to the release of pro-inflammatory cytokines and cell lysis [11]. The GSDMD-N fragment binds to inner-leaflet phosphoinositides and cardiolipin, then forms large transmembrane pores made of about 16 protomers. These pores have an inner diameter of roughly 10 to 14 nm, which is wide enough for mature IL-1β (about 4.5 nm) and IL-18 to pass through, but still small enough to keep larger cytosolic proteins inside until the membrane finally ruptures [11–13]. The assembly of GSDMD pores is regulated by the types of membrane lipids present, ESCRT-mediated membrane repair, and the Ragulator–Rag–mTORC1 pathway, which controls GSDMD-N oligomerization and sets the threshold for pyroptosis to occur [14, 15]. Besides, causing cell lysis, sublytic GSDMD pores expose phosphatidylserine on the cell surface and activate the coagulation cascade via tissue factor, linking pyroptosis to the increased blood clotting observed in endotoxemia and sepsis [16]. The key role of GSDMD-NT is highlighted by recent studies showing that directly blocking the GSDMD pore, without interfering with upstream inflammasome assembly, caspase-1 activation, or pro-IL-1β processing, can delay pyroptosis, reduce IL-1β release, and improve survival in models of LPS- and CLP-induced sepsis [15]. Currently, we can distinguish between several pathways of pyroptosis activation. However, these processes share a common outcome: the processing of IL-18 and IL-1β, the activation of GSDMD, and ultimately the rupture of the cell membrane, releasing IL-18 and IL-1β [11, 12]. Both cytokines are synthesized as biologically inactive precursors (pro-IL-1β, pro-IL-18) and become active only following cleavage by inflammatory caspases. They lack a classical secretory signal peptide and are released primarily through GSDMD pores, with complete release occurring during terminal membrane rupture [17]. After entering the extracellular space, IL-1β binds to IL-1R1, promoting neutrophil recruitment, endothelial activation, and Th17 polarization. In contrast, IL-18 acts in concert with IL-12 to stimulate IFN-γ production by NK and T cells, thereby involving local pyroptotic events in systemic innate and adaptive immune responses [17].

### The canonical pathway of pyroptosis

**Fig. 1:**
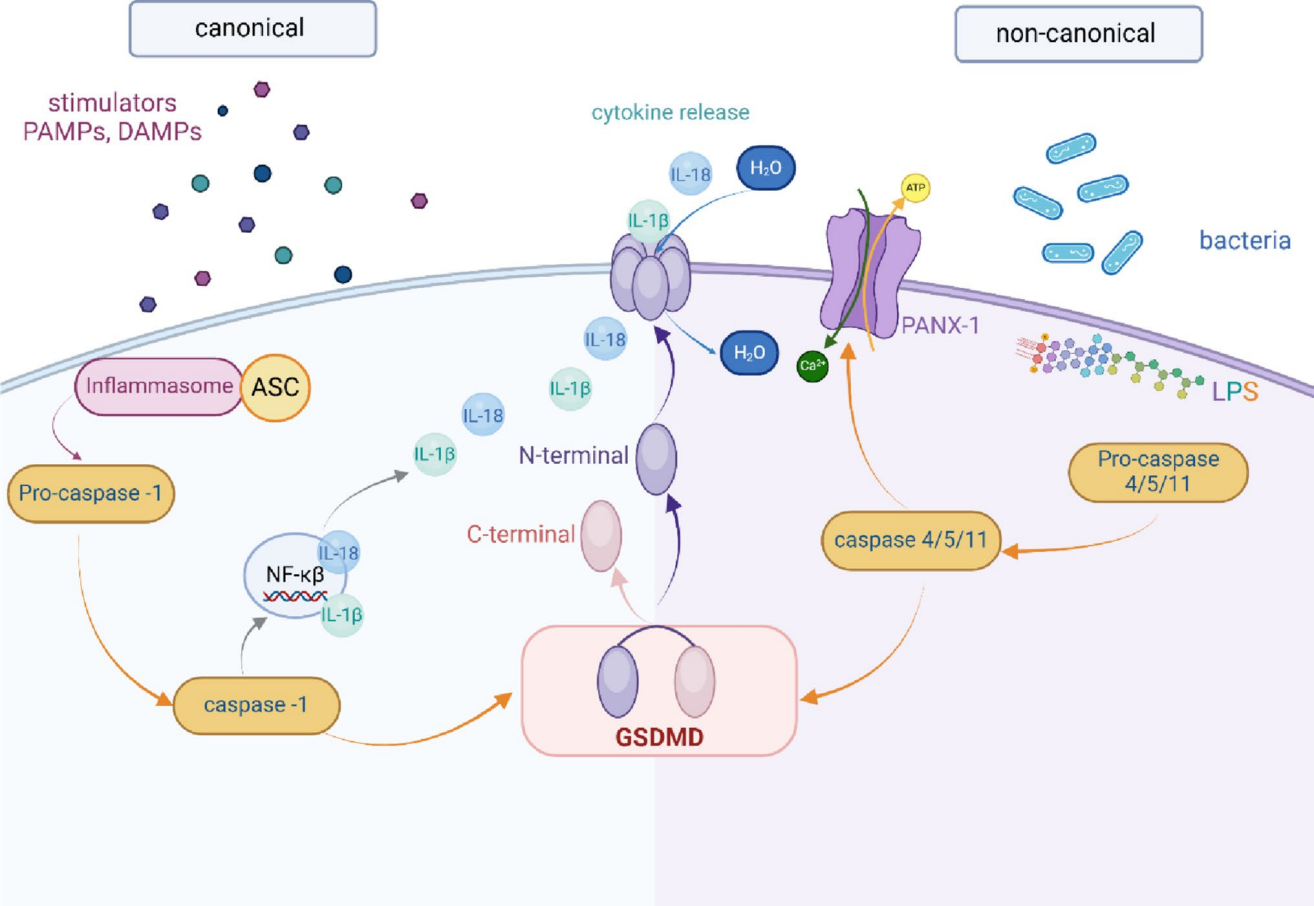
Comparison of canonical and non-canonical pyroptosis pathways. The canonical pathway is initiated by pathogen-associated molecular patterns (PAMPs) and damage-associated molecular patterns (DAMPs), activating inflammasomes and caspase-1, leading to the cleavage of gasdermin D (GSDMD) and the release of cytokines (IL-1β, IL-18). In contrast, the noncanonical pathway is triggered by intracellular lipopolysaccharides (LPS) from bacteria, activating caspases 4, 5, or 11, directly cleaving GSDMD without inflammasome formation. Both pathways ultimately result in cell membrane pore formation and cytokine release, culminating in pyroptotic cell death (Created in BioRender. Kulbacka, J. (2025) https://BioRender.com/k72h230)

One of the canonical pathways of pyroptosis is initiated by activating pattern recognition receptors (PRRs) (Fig. 1). The canonical pathway identifies not only PAMPs and DAMPs but also cytoplasmic disruptions, which have recently been referred to as homeostasis-altering molecular properties (HAMPs). The activation of PRRs leads to the assembly of inflammasomes, which are multiprotein complexes. They are made of sensor proteins: Absent in Melanoma 2 (AIM2)-like receptor (ALR) or leucine-rich repeat (LRR) – containing receptors and nucleotide-binding domain (NBD), the second component is pro-caspase-1, and the third is an adaptor protein (ASC) [18].

**Membrane-bound PRRs** that we can distinguish are C-type Leptin Receptors (CLRs) and Toll-like receptors (TLRs). TLRs can recognize Lipopolysaccharides (LPS), which are bacterial toxins, mainly the lipid A fragment. TLRs prime specific PRRs, thereby augmenting immune responses [19]. CLR molecules can trigger signaling pathways that directly activate nuclear factor-κB, which facilitates the NLRP3 signal, while other CLRs impact signaling through TLRs [20].

**Cytosolic-PRRs.** NOD-like receptors (NLRs) or ALRs, which recognize PAMPs or DAMPs released during infection or cellular damage. Other receptor proteins, such as Retinoic acid-inducible gene I (RIG-I)-like receptors (RLRs), can also initiate inflammasome assembly and induce pyroptosis in response to viral infection [21, 22]. Canonical inflammasomes are multimeric cytosolic platforms built around three core modules: a sensor PRR (e.g., NLRP1, NLRP3, NLRC4, AIM2), the adaptor protein ASC (apoptosis-associated speck-like protein containing a CARD), and the effector pro-caspase-1. Upon sensor activation, exposed pyrin domains (PYD) of the receptor nucleate ASC oligomerization through homotypic PYD–PYD interactions, generating the characteristic ~ 1 μm “ASC speck”. The CARD domain of ASC then recruits pro-caspase-1 via CARD–CARD interactions, driving its proximity-induced autoproteolytic maturation into the active p20/p10 heterotetramer, which in turn cleaves pro-IL-1β and pro-IL-18 into their bioactive forms and processes gasdermin D (GSDMD) to trigger pore formation and pyroptotic lysis [22]. It has been demonstrated that several NLRs can activate the formation of inflammasomes; among those NLRs, we can distinguish NLR family pyrin domain-containing protein 1 (NLRP1) - a receptor that can detect bacterial toxins, such as anthrax lethal toxin or Toxoplasma gondii, and form NLRP1 inflammasomes [23]. Other NLRs, such as NLRC4, recognize both the type III and type IV secretion systems and bacterial flagellin, activating the inflammasome and leading to IL-1β and IL-18 release and pyroptosis [24]. NLRP6 and NLRP12 are also involved in recognizing microbial infections and inflammatory stimuli. They also regulate the inflammatory process and pyroptosis induction by forming inflammasome complexes or degrading NF-κB-inducing kinase (NIK), thereby exerting negative regulation. Reduction of NIK impairs NF-κB signaling [5, 25].

One of the best-studied NLRs is NLRP3. Its response can be triggered by a diverse array of stimuli, including bacterial and viral infections, uric acid crystals, extracellular ATP, and environmental pollutants [26]. NLRP3 activation classically requires two signals: a priming step (signal 1), in which TLR/NF-κB engagement upregulates NLRP3 and pro-IL-1β transcription, and an activation step (signal 2), provided by cellular stress events such as K⁺ and Cl⁻ efflux, Ca²⁺ mobilization, lysosomal destabilization, or mitochondrial ROS, which licenses NLRP3 oligomerization and recruitment of ASC and pro-caspase-1 [18, 20]. The NLRP3 inflammasome then activates caspase-1 to produce mature cytokines, IL-1β/IL-1. The interaction between bacteria and the inflammasome in macrophages involves M. tuberculosis, which triggers phagosomal damage via the ESX-1 pathway, leading to K+ efflux and subsequent NLRP3 inflammasome activation. Despite this, M. tuberculosis can also inhibit the NLRP3 inflammasome through the action of the bacterial phosphokinase PknF [27]. Studies have shown that AIM2 differs from other NLR family proteins because it responds to cytosolic double-stranded DNA by triggering the assembly of the AIM2 inflammasome, thereby activating caspase-1. AIM2 uses its HIN200 domain to bind dsDNA and its PYD to recruit ASC, illustrating that the sensor–ASC–caspase-1 scaffold is conserved across both NLR- and ALR-type inflammasomes. This occurs without activating other inflammatory molecules, such as NLRs or TLRs [28, 29].

### Noncanonical pathway of pyroptosis

The noncanonical pathway of pyroptosis (Fig. 1) is activated by intracellular lipopolysaccharides (LPS), cell wall components, cholera Toxin B, and Gram-negative bacteria (i.e., *Escherichia coli*) [30, 31]. The caspase activation and the recruitment domain (CARD) of cytosolic pro-Caspase-11/4/5 bind to the lipid A of LPS. This causes conformational changes in pro-caspases 4/5/11, leading to oligomerization, limited auto-proteolysis, and GSDMD cleavage, thereby inducing pore formation. This serves as a secondary signal to induce pyroptosis within the cell [17]. Activated caspase-11 can cleave Pannexin-1. As a result, ATP is released, and cell death can be executed through P2 × 7R-related pyroptosis [32]. It is important to note that intracellular LPS can also cause abnormal hyperactivation of caspase-11, which becomes harmful during endotoxic shock [17]. The noncanonical pathway has also been implicated in metabolic diseases associated with mitochondrial dysfunction [14]. During the noncanonical pathway, inflammatory molecules such as ATP, HMGB1, IL-18, and IL-1α are liberated from the cytosol and affect the production of inflammatory cytokines such as IL-1β and IL-18 [33]. IL-1α is directly cleaved by caspase-5 and caspase-11, whereas IL-18 is directly cleaved by caspase-4 and caspase-11 [34]. Detection of bacterial RNA by NLRP3 and LPS binding to procaspase-11 leads to an interaction between procaspase-11 and NLRP3, forming the inflammasome, which triggers caspase-1 activation [35, 36]. Caspase-4 and 11 are expressed extensively in various non-monocytic cells, such as numerous epithelial and endothelial cells and keratinocytes [37]. Activated caspases-4/5/11 can initiate the creation of NLRP3 inflammasome. Furthermore, studies have demonstrated that caspase-4 can cleave both IL-18 and caspase-7 - the executioner caspase [41]. The efflux of K+ caused by caspase-11 and membrane rupture can also trigger activation of the NLRP3 inflammasome, resulting in cleavage of pro-IL-1β and pro-IL-18 [46]. Human caspase-4 is unique among caspases. Bacteria like Yersinia and Francisella reduce lipid A acylation to avoid TLR4 recognition. Caspase-4, compared to caspase-11, has an exceptional ability to recognize the underacylated lipid A [39]. Some pathogens, such as *Salmonella*, can induce pyroptosis by injecting effector proteins that activate inflammasomes in canonical and noncanonical pathways with caspase-4/11 [40]. Others, such as Shigella, can prevent caspase-4/11 activation by ADP-ribosylating arginine residues, including OspC3 [38, 41]. Recent investigations have discovered that caspase-8, an apoptotic caspase, is a crucial factor in the transition to pyroptosis when apoptosis and necroptosis are blocked. This provides an alternate method of pyroptosis activation. Caspase-8 can also cleave GSDMD, probably at the same site as caspase-4/5/11 [46; 45]. Pyroptosis can also be triggered by inflammasome-independent mechanisms. For example, CAR-T cells can induce pyroptosis in target tumor cells via granzyme B-mediated caspase-3 activation and GSDME cleavage. Similarly, cytotoxic lymphocytes use granzyme A to activate GSDMB and granzyme B to activate GSDME, thereby inducing pyroptosis, membrane rupture, and release of inflammatory mediators, independent of caspase-1 or inflammasome sensors [42–46]. These pathways highlight the variety of mechanisms that activate gasdermin and extend the concept of pyroptosis beyond inflammasome involvement.

### Regulators of canonical and non-canonical pyroptosis

An increasing number of studies have demonstrated that pyroptosis is not only dependent on inflammasome activation but also tightly regulated by transcription factors and other modulators that fine-tune canonical and non-canonical pathways. For instance, interferon regulatory factor 1 (IRF1) has been shown to promote AIM2-mediated pyroptosis by facilitating inflammasome assembly [47], while IRF8 acts as a positive regulator of NLRC4-driven pyroptosis in macrophages [47]. Similarly, immunity-related GTPase family member B10 (IRGB10) exerts dual control over both NLRP3- and AIM2-mediated inflammasome activation, thereby bridging microbial sensing with execution of pyroptotic death [48]. Recent findings have also expanded the role of caspases beyond their classical functions, as caspase-6 was identified as a regulator of NLRP3 inflammasome activation and pyroptosis [49, 50]. These observations complement current knowledge that inflammasome signaling integrates multiple cellular stress signals, such as RIG-I–mediated sensing [51], or metabolic modulators, including itaconate and succinate, which affect NLRP3 activity and GSDMD cleavage [52]. Taken together, these regulators demonstrate that pyroptosis is controlled at several hierarchical levels, i.e., transcriptional, enzymatic, and metabolic, underlining its role in host defense, inflammation, and cancer biology.

### Techniques for measuring pyroptosis activation

Measuring caspases and other markers can determine if a cell has undergone pyroptosis or another type of PCD. Various techniques can be employed to measure the levels of those molecules. Flow cytometry is a commonly used method for analyzing the properties of individual cells, including both physical and chemical characteristics. It can detect alterations in the cell membrane, such as the exposure of phosphatidylserine, a hallmark of early apoptosis [16]. Western blotting, a widely used method for detecting the expression and activation of specific proteins, can be used to identify proteins such as caspases. This method involves separating protein samples by size and charge using electrophoresis. Incubation with antibodies that target the proteins of interest is then used to probe the membrane to determine their activation state and expression levels. GSDMD cleavage yields the N-terminal form (GSDMD-N), which can be detected by Western blotting with an antibody against the N-terminal region of GSDMD. Other proteins that can serve as markers of pyroptosis include caspase-11 and IL-1β. Those molecules can be detected using antibodies against GSDMD, caspase-11, and IL-1β [53, 54]. Immunofluorescence microscopy is a method that involves attaching fluorescent tags to proteins of interest and imaging cells with a fluorescence microscope to visualize specific proteins and cellular structures. It can be used to visualize caspase activation and other indicators of apoptosis or pyroptosis. This method can localize GSDME diffusion in the cytoplasm or at the membrane during pyroptosis [55]. MAP2/GSDMD colocalization can also be used to determine whether a cell is undergoing pyroptosis [56]. Another, but indirect, method is to measure the secretion of inflammatory cytokines such as IL-1β or IL-18. It was also noted that TAK1 (transforming growth factor-β-activated kinase 1), a MAPKKK family serine/threonine kinase, mediates cytokine-induced inflammatory signaling via the NF-κB and MAPK pathways [57]. Effective techniques, such as LDH and PI staining, are used to assess the extent of pyroptosis [58]. Since LDH release, PI uptake, and cytokine secretion can also occur during apoptosis, necroptosis, or ferroptosis, it should be noted that none of these biochemical or morphological assays are completely specific to pyroptosis. Therefore, genetic methods such as CASP1 or GSDMD/GSDME knockout or knockdown, in combination with repair experiments and confirmation of gasdermin N-terminal cleavage and caspase-1 activity, are necessary for the definitive identification of pyroptosis [59]. These techniques remain the best way to distinguish pyroptosis from other types of cell death.

## Pyroptic signaling

### Pyroptosis-related genes

Recent studies have described 33 genes associated with pyroptosis [60]. Five of them, NLRP1, NOD1, NLRC4, CASP9, and PLCG1, in addition to being used in lung adenocarcinoma diagnosis, may be part of its treatment [61]. Pyroptosis is intrinsically linked to inflammasome formation. The functions of the various proteins encoded by the mentioned genes have been determined. The first gene encodes the NLRP1 protein. The molecule is responsible for forming the inflammasome complex and activating caspase-1. Similarly, the NLRC4 protein constitutes a building element of the inflammasome [62]. The protein encoded by the NOD 1 gene acts as a pattern recognition receptor and is positioned in the initiation of inflammation [63]. Lastly, PLCG1 (phospholipase C, gamma 1) catalyzes the formation of inositol 1,4,5-trisphosphate and phosphatidylinositol 4,5-bisphosphate diacylglycerol, which are important in the intracellular transduction of tyrosine kinase activators [64]. The prognostic values of pyroptosis-related genes were also assessed for patients with ovarian cancer. The study identified seven genes that can predict overall survival among patients with this cancer [65]. One of those markers is caspase-3, which cleaves the non-syndromic hearing impairment protein (DFNA5) after Asp270. It leads to the formation of a necrotic DFNA5-N fragment that translocates to the plasma membrane, permeates it, and induces pyroptosis. GSDMD-associated DFNA 5 belongs to the gasdermin family and causes apoptotic cells to progress to secondary necrosis [66]. It is important that inhibition of caspase-3 activity after radiotherapy or chemotherapy inhibits angiogenesis, potentially leading to therapeutic benefits [67]. Caspase-8 activates caspase-3 and can regulate DFNA5-induced pyroptosis [68]. It is also capable of cleaving some substrates at the same site as caspase-1/11, so its potential to directly activate GSDMD is currently under investigation. It has also been proven to be involved in inflammatory processes by processing IL-1β [69]. The literature also highlights the importance of another gasdermin family protein (GSDME) in programmed lytic cell death. Small interfering RNA in SCC7 cells inhibits GSDME expression, significantly increasing tumor markers such as CD44 and ALDH1. Studies are emerging that prove the use of apoptin to indicate GSDME cleavage. They depend not only on activated caspase-3 but also on caspase-9. Expression of this proline-rich apoptin structural protein in cancer cells by oncolytic adenoviruses may provide a tumor-specific method for clinical cancer therapy [70].

Further research using caspase-9 may make this protein a therapeutic target not only for cancer but also for degenerative and developmental diseases [67]. It has been reported that cellular metabolism, such as the Krebs cycle, can impact pyroptosis. In this relationship, changes in metabolite concentrations may serve as biomarkers of pyroptosis. Studies indicate that succinate inactivates gasdermin D, thereby inhibiting pyroptosis. In contrast, itaconate, which blocks the action of succinate dehydrogenase, also modifies NLRP3 to inhibit inflammasome activation [71, 72, 73]. It should also be noted that pyroptosis cannot be characterized solely by gene expression, as it is a type of cell death carried out at the post-translational level by proteolytic cleavage of gasdermins. While epigenetic silencing of GSDME inhibits chemotherapy-associated pyroptosis until its expression is restored, studies on function in cancer confirm that genetic ablation of CASP1 or GSDMD suppresses pyroptotic lysis [74]. The best method for pyroptosis identification is still functional validation, not expression profiling, as more recent research demonstrates that loss- or gain-of-function of gasdermins influences tumor progression and response to therapy [75, 76].

### Role of caspase-1 in pyroptosis

Inflammasomes cause caspase-1 activation in response to multiple stimuli [77]. The substrate for inflammatory caspases and also a critical pyroptotic mediator is gasdermin D (GSDMD). Active caspase-1 and 4, 5, and 11 cleave GSDMD in the region between the N-terminal and C-terminal domains, which releases the gasdermin-N domain. The free N-terminal domain then binds to phosphatidylinositol in the plasma membrane and forms pores in the cell membrane, causing lytic cell death [78]. In neutrophils, GSDMD processing is not affected by caspase proteins but depends on ELANE (neutrophil-specific serine protease, neutrophil elastase) [79]. The ELANE pyroptosis-related gene can promote invasion and metastasis in clear cell renal cell carcinoma, making it a potential prognostic and therapeutic marker for patients with clear cell renal cell carcinoma [80]. Cleaved GSDMD selectively binds to plasma membranes containing lipids such as mitochondrial and bacterial lipid cardiolipin and phosphatidylinositol phosphates, which are found on the inner leaflet of mammalian cell membranes [13]. Pyroptosis causes the release of DAMPs, including high-mobility group box 1 (HMGB1) and lactate dehydrogenase (LDH), which potentiate inflammation [81]. As a result of the described events, interleukin-1β (IL-1β) and − 18 (IL-18) are released from the resulting pores and lead to a strong inflammatory response. Caspase-1 can be activated in macrophages infected with bacteria such as *Salmonella* or *Shigella*. Through the protease, the proforms of inflammatory cytokines, IL-1β and IL-18, are converted into active forms. The consequence of their activation is host cell death [4]. In summary, pyroptosis can be identified by a variety of markers, including activation of caspases-1, −4, −5, and − 11, GSDMD cleavage, and release of IL-1β and IL-18 [82].

## Pyroptosis versus other forms of cell death

Apoptosis, necroptosis, and pyroptosis are the three most widely used and best researched processes of programmed cell death. Although they appear to be similar processes, they have different morphologies, and other effector proteins are involved [83]. These different pathways, which are programmed cell death, can sometimes overlap. Pyroptosis is an inflammatory form of cell death that depends on caspases 1, 4, 5, and 11. It is marked by pore formation mediated by gasdermin, cytokine release, and pyroptotic body formation, which are vesicle-like structures distinct from apoptotic bodies. Apoptosis, on the other hand, is a process that doesn’t cause inflammation and involves initiator (caspase-8, −9, −10) and executioner caspases (caspase-3, −6, −7). This process causes membrane blebbing, the formation of apoptotic bodies, and DNA fragmentation. Necroptosis is a controlled, caspase-independent cell death pathway that involves RIPK1/RIPK3/MLKL and causes cells to swell and the plasma membrane to break. Ferroptosis is an inflammatory process that does not include caspases. It is caused by excessive iron and lipid peroxidation, and can be induced by RSL3, erastin, or acrolein [84]. Unlike pyroptosis, ferroptosis features distinct mitochondrial changes and oxidative lipid damage as execution mechanisms initiated by perturbations in lipid redox homeostasis and iron handling [84–86]. Mechanistically, these two inflammatory forms of cell death, i.e., pyroptosis and ferroptosis, are increasingly recognized to interconnect via autophagy and related stress signaling, which may switch between death pathways depending on the stimuli and context, especially in cancer and chronic inflammatory disease settings [86]. In the table below, we present the similarities and differences between the most common forms of cell death in multicellular organisms. Table 1 below summarizes and compares the described cell deaths.

**Table 1:** Comparison of key features of pyroptosis, necrosis, or apoptosis

Subject	Pyroptosis	Necroptosis	Apoptosis	Ferroptosis
Inflammation [85, 87]	present	present	no	Regulated
Type of cell death [85]	programmed cell death (inflammatory)	regulated necrosis, caspase-independent	programmed cell death (non-inflammatory)	regulated necrotic cell death (iron-dependent, lipid peroxidation–driven)
Reason for cell death [88]	infection with intracellular pathogens, PAMPs/DAMPs, and inflammasome activation	death receptor stimulation (TNFα, TRAIL, FasL), TLR ligands, IFNs, metabolic stress; requires RIPK1–RIPK3–MLKL axis	abnormalities in cell function and control of cell division rate, DNA damage,	lipid ROS accumulation, iron overload, GPX4 inhibition; triggers include RSL3, erastin, acrolein, sorafenib, cystine deprivation
Changes in the nucleus [89]	DNA damage, chromatin condensation (partial), intact or fragmented DNA	intact nucleus dehydration of the nucleus, or chromatin condensation or fragmentation	DNA damage chromatin condensation fragmented nucleus destruction of the nuclear membrane ordered fragmentation of DNA	intact nucleus; no chromatin condensation or fragmentation
Intracellular changes [83, 90, 91]	pore formation by GSDMD/E; cell swelling; membrane rupture; release of IL-1β/IL-18	cell swelling; organelle swelling; MLKL-mediated membrane permeabilization; loss of ion homeostasis	cell shrinkage, membrane blebbing, apoptotic body formation	smaller mitochondria with dense membranes; reduced cristae; outer mitochondrial membrane rupture; lipid peroxide accumulation
Characteristic structures [91]	pyroptotic bodies (vesicle-like, 1–5 μm) [92]	None	Apoptotic bodies (1–5 μm)	none
Activated caspases [91, 93, 94]	caspase-1, −4, −5, −11 (plus caspase-3, −8 in crosstalk)	none (caspase-8 inhibits by blocking RIPK1/RIPK3)	caspase-3, −6, −7 (executioners); caspase-8, −9, −10 (initiators)	none (caspase-independent)
Key mediators [89, 91]	inflammasomes (NLRP3, AIM2, NLRC4), caspase-1, GSDMD/E	RIPK1, RIPK3, MLKL (phosphorylation-driven)	Bcl-2 family, cytochrome c, Apaf-1, caspase cascade	iron, lipid ROS, GPX4 inhibition, ACSL4, system Xc− inhibition
Markers and released mediators [91]	IL-1β, IL-18, HMGB1, LDH	DAMPs (ATP, HMGB1), ROS, phosphatidylserine exposure	cytochrome c, DNA laddering, PARP cleavage	lipid peroxides, malondialdehyde (MDA), 4-hydroxynonenal (4-HNE), oxidized phospholipids
Detection methods [84, 89, 91]	caspase-1 activity, GSDMD cleavage, IL-1β/IL-18 ELISA, LDH release, PI staining, genetic KO (Casp1/Gsdmd)	MLKL phosphorylation, RIPK1/RIPK3 detection, necrosulfonamide/nec-1 sensitivity, genetic KO (Ripk3, Mlkl)	Annexin V, TUNEL, caspase activity assays, DNA fragmentation, Comet assay	lipid peroxidation probes (BODIPY-C11), MDA/4-HNE assays, GPX4 activity, rescue with ferroptosis inhibitors (ferrostatin-1, liproxstatin-1), iron chelators

**PI** - propidium iodide, **EtBr** - ethidium bromide

Recent studies also describe PANoptosis, a highly inflammatory form of programmed cell death that combines features of pyroptosis, apoptosis, and necroptosis, under the control of a multiprotein complex known as the PANoptosome. To provide the integration, innate immune sensors such as ZBP1, AIM2, and NLRC5 are necessary and detect, e.g., pathogen- or stress-derived signals, recruiting caspase-8, RIPKs, and inflammasome components to orchestrate coordinated death signaling [95, 96]. Panoptosis allows cells to avoid pathogen-induced blockade of individual death pathways (e.g., pyroptosis alone) by activating a redundant, synergistic response that enhances pathogen clearance and host defense, a notion supported by recent mechanistic reviews of programmed cell death networks [95, 97].

## Implication on immune checkpoints of pyroptosis

Pyroptosis has a dual role in cancer: the inflammatory response and inflammatory factors generated during this form of programmed cell death can stimulate the transformation of normal cells into tumor cells. It is also involved in creating a tumor microenvironment, promoting its growth and progression. On the other hand, it can lead to tumor cell death, and with proper stimulation, it can significantly suppress cancer [98]. Chronic pyroptosis-driven release of IL-1β and IL-18 sustains a persistent inflammatory environment that promotes tumor initiation and progression. This pattern has been documented in gastric cancer, where pyroptosis-related signatures correlate with adverse immune microenvironment features [99], as well as in lung adenocarcinoma and ovarian cancer, where dysregulated expression of pyroptosis effectors is associated with poorer prognosis [61, 65]. In contrast, robust pyroptosis induction in tumor cells exerts potent antitumor effects across multiple cancer types. For example, GSDME-dependent pyroptosis induced by triptolide suppresses head and neck squamous cell carcinoma [55], apoptin-driven GSDME cleavage promotes pyroptosis in colorectal cancer cells [70], and α-NETA elicits caspase-4/GSDMD-mediated pyroptosis in ovarian cancer, resulting in decreased tumor volume and weight in vivo [100]. The therapeutic significance of this pathway is further highlighted by evidence that GSDME-mediated pyroptosis of target cells by CAR-T cells drives cytokine release syndrome, thereby directly linking pyroptosis to immune-effector-cell-based cancer therapy [42, 46]. It was found that pyroptosis, occurring in approximately 15% of tumor cells, was sufficient to eliminate the entire 4T1 mammary tumor implant [101]. Pyroptosis can be detected in cells by measuring intracellular markers, such as caspases 1/3/4/5/8/11, granzymes, the GSDM family, and cell-released substances HMGB1, ATP, IL-1β/18, and lactate dehydrogenase. These can be examined using ELISA. Western blotting is performed to determine caspase-1 and gasdermin D cleavage, which is sufficient to initiate pyroptosis [102, 103]. LDH is a readily available marker for detecting pyroptosis. However, it is insufficient to distinguish this process from other forms of programmed cell death, as LDH is also released during necrosis and apoptosis [104]. Therefore, the best way to detect this cell death is to follow the levels of a couple of substances involved with the process. Expression levels of caspase-1, IL-1β, and IL-18 were assessed to investigate the pro-inflammatory role of pyroptosis induced by alcohol accumulation in the development of alcohol esophagitis. The levels of these three markers were studied using qRT-PCR and western blotting, and were found to correlate with the inflammatory process underlying esophagitis [105]. IL-18, GSDMD, and caspase-4 were measured by ELISA and Western blot to demonstrate that α-NETA treatment induces pyroptosis in ovarian cancer cells. To confirm the efficiency of those markers in detecting pyroptosis, the samples were investigated with an electron microscope, where the typical pattern of cell lysis, membrane rupture, and cytoplasmic leakage was observed. As a result of the study, the application of α-NETA treatment decreased tumor volume and weight [100].

Another useful method is scanning electron microscopy (SEM), which can distinguish cells undergoing pyroptosis from those killed during necrosis or apoptosis. SEM showed that during necrosis, cells exhibited a spherical shape with protruding, bursting extensions. Pyroptotic cells exhibited less swelling than cells that underwent plasma membrane rupture and generated several bubble-like protrusions. These data together demonstrated that GSDMD-N forms oligomers during pyroptosis [106]. Recent studies have analyzed and identified the connection between pyroptosis-related genes and tumor characteristics, including tumor progression, patient prognosis, and response to immunotherapy. Based on the expression of pyroptosis-related regulators, predicting the overall survival of patients with tumors like glioma, thyroid cancer, and breast cancer was possible [99]. The creation of a pyroptosis-related risk signature enabled classification of patients with thyroid cancer into low- and high-risk groups based on expression of 4 pyroptosis-related genes: IL18, GSDMC, PJVK, and NOD1. The low-risk groups were associated with higher expression of IL18 and NOD1 genes. According to the ssGSEA analysis, this group demonstrated higher immune activity overall and better survival. The high expression of GSDMC and PJVK was associated with poorer survival. Expression of common immune checkpoints, including PD-1, PD-L1, PD-L2, CTLA4, TIGIT, and TIM-3, was significantly higher in patients in the low-risk group, suggesting that low-risk patients may respond better to checkpoint inhibitor therapy [107]. Hiroaki et al. also observed the antitumor activity of IL18. They presented that IL-18 promoted the expansion of APC-like NK cells derived from patients with lung cancer. Those cells effectively killed the lung cancer cell line PC-9 through antibody-dependent cell-mediated cytotoxicity (ADCC) [108].

The same classification, based on gene expression of IL1B, CASP8, TP53, PYCARD, IL18, and IL6, was performed to study patients with glioma. Patients were divided into two clusters: the patients in cluster 1 had significantly higher overall survival than those in cluster 2. The analysis between clusters showed that samples with worse prognoses had higher activity of immune pathways. The complement and coagulation cascades, ECM receptor interactions, and antigen processing were more prominently studied in this group. This group also had a higher level of immune cell infiltration, composed of dendritic cells, B cells, macrophages, and CD8 + T cells [109]. This study and a study by Yu Zeng and colleagues concluded that pyroptosis-based classification can be used to identify a tumor phenotype [110]. J.B. Wu and Y. Zhu presented a concept, similar to the previous ones, of a PyroptosisScore to quantify the pyroptosis pattern in breast cancer. The study proved that this score based on pyroptosis-related gene expression could effectively predict the prognosis of patients with BCRA and their immunotherapy response. Patients with a low PyroptosisScore exhibited elevated expression of immune checkpoints and greater infiltration of immune cells into the microenvironment. Moreover, they had higher sensitivity to immunotherapy than those with a high PyroptosisScore [111]. All of the above classifications were constructed by evaluating the mRNA levels of known pyroptosis-related genes in tumor and normal tissues, and then screening those genes for differential expression. The construction and analysis of PPI networks were used to select the most relevant genes for creating these classifications [109, 111].

As was demonstrated, expression-based pyroptosis patterns have been correlated with patient prognosis and immune infiltration patterns across various cancers; however, these findings necessitate careful interpretation. Pyroptosis occurs via caspase- and gasdermin-dependent cleavage events; therefore, definitive proof of pyroptotic activity requires functional or genetic evidence, such as CASP1 or GSDMD knockout or restoration of silenced GSDME. Consequently, the most reliable studies combine expression data with genetic modifications or cleavage assays, thereby confirming that the identified immune checkpoint correlations indeed indicate pyroptosis rather than unrelated inflammatory processes [75, 112].

## Computational approaches to pyroptosis identification

With the multiplicity markers and differentiation between various cell death types, machine learning and neural network approaches have shown promise in refining our understanding and detection of pyroptosis beyond traditional molecular assays. For example, pyroptosis-associated gene profiles in ischemic stroke have been identified using integrated machine learning models such as LASSO (Least Absolute Shrinkage and Selection Operator), RF (Random Forest), and SVM (Support Vector Machine), which have revealed a core of differentially expressed PRDEGs [113]. Deep learning models based on genes associated with pyroptosis have successfully differentiated between latent and active TB (tuberculosis) cases in infectious disease settings [114]. Similarly, pipelines that combined WGCNA (Weighted Gene Co-expression Network Analysis) with several machine learning algorithms discovered seven hub pyroptosis genes associated with the immune landscape and the progression of Alzheimer’s disease [114, 115]. Furthermore, morphology-based signatures suggestive of pyroptotic cell death have been identified using machine learning applied to image-based detection [116]. In addition to advancing classification and biomarker discovery, AI is transforming the rational targeting of pyroptosis effectors. Transformer-based DL models, trained on protein-protein interaction interfaces from the Protein Data Bank and integrated with Rosetta FastDesign sequence optimization, have recently enabled the de novo design of peptide binders targeting the previously considered “undruggable” smooth surface of GSDMD-NT. This approach has produced the lead peptide SK56, which inhibits GSDMD pore conductance, promotes ESCRT-mediated membrane repair, and reduces inflammatory damage in vivo [15]. These methods provide strong, high-throughput strategies to complement functional and genetic assays for pyroptosis in various biological contexts, although they are model-dependent and require validation.

## Clinical applications: current challenges and limitations

Although therapeutic interest in pyroptosis is increasing rapidly, several challenges continue to impede its clinical translation. The dual nature of pyroptosis is a primary concern: it exerts antitumor effects when robustly induced in malignant cells, but promotes tumorigenesis when chronically sustained at sub-lytic levels within the tumor microenvironment. Thus, the pathway may suppress or facilitate disease progression, depending on the intensity of activation, cellular context, and tissue type [9, 49, 95]. This narrow therapeutic window is further complicated by cytokine release syndrome associated with pyroptosis, as observed during GSDME-mediated cytotoxicity by CAR-T cells, highlighting the risk of on-target systemic toxicity [42, 43]. Additionally, the pharmacological toolkit remains underdeveloped. Most current modulators, including disulfiram-like GSDMD inhibitors, NLRP3 inhibitors, and natural pyroptosis inducers such as triptolide and α-NETA, exhibit limited specificity, poor bioavailability, off-target effects on other caspase and gasdermin family members, and lack validated delivery strategies for tumor-selective activation [53, 56, 103]. Furthermore, there are no pyroptosis-specific clinical biomarkers. Commonly used assays to detect LDH, IL-1β, and IL-18 overlap with apoptosis, necroptosis, and broader inflammatory states. Therefore, definitive evidence of pyroptotic activity in patient samples currently requires combined cleavage assays (caspase-1/GSDMD) and, ideally, genetic confirmation (CASP1/GSDMD knockout or GSDME restoration), which are challenging to implement clinically [73, 82, 101, 111]. Finally, while pyroptosis-related gene signatures and machine-learning classifiers demonstrate potential for prognostic stratification and prediction of immunotherapy response in various cancers, including glioma, breast, thyroid, lung, and ovarian [58, 59, 63, 106, 108–110], these models are typically retrospective, derived from heterogeneous transcriptomic cohorts, and lack prospective validation. Their generalizability and clinical utility remain to be established in dedicated trials [73, 111]. Addressing these limitations will require the development of cell- and tissue-selective gasdermin modulators, the integration of multi-omic and functional readouts into pyroptosis-specific companion diagnostics, and the prospective clinical evaluation of pyroptosis-based stratification tools.

## Summary

**Fig. 2:**
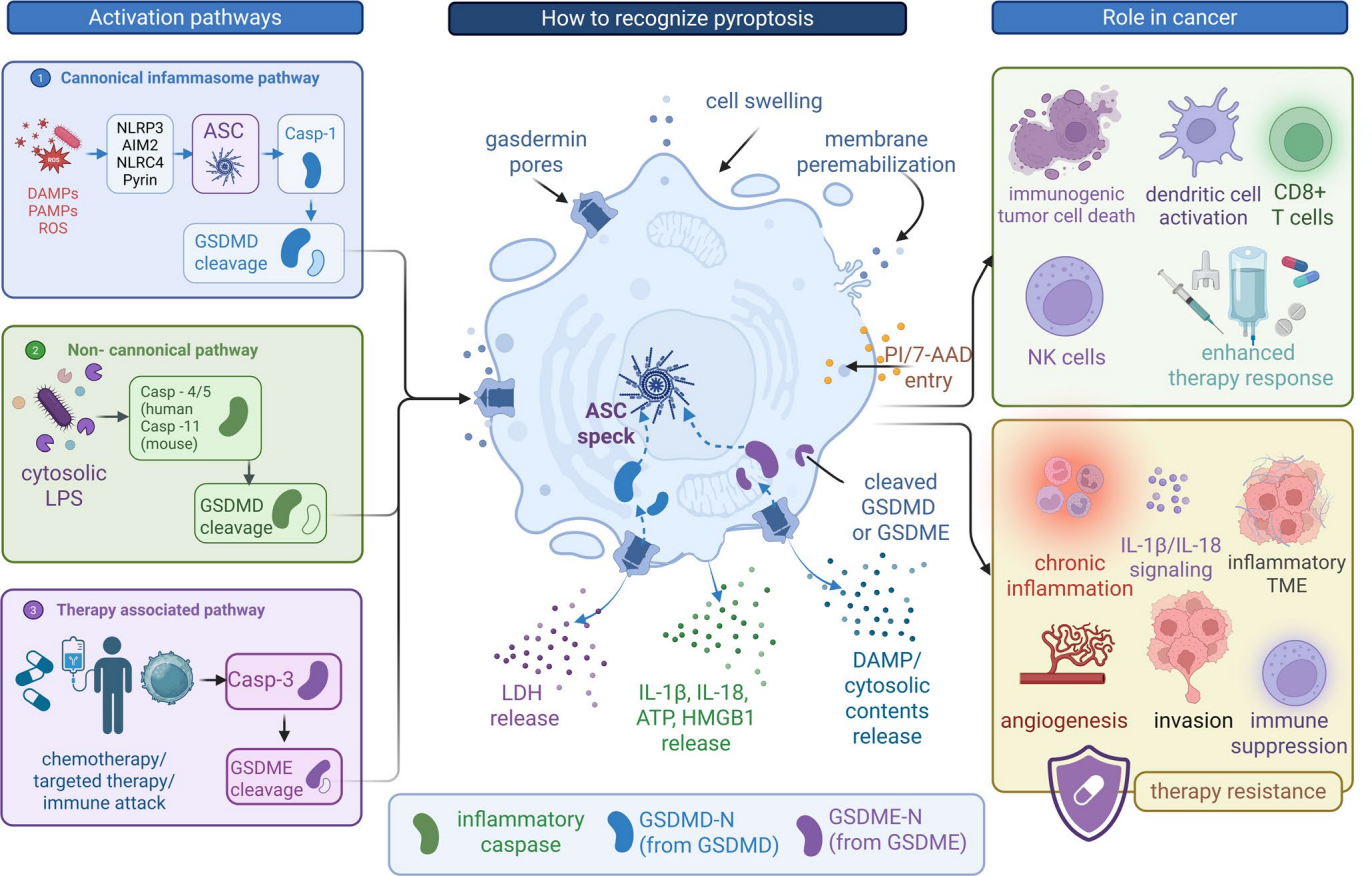
Summary of pyroptosis signaling pathways and their role in cancer. Pyroptosis can be initiated through canonical inflammasome activation, non-canonical cytosolic LPS sensing, or therapy-associated caspase 3-mediated activation. These pathways converge on gasdermin cleavage, mainly GSDMD or GSDME, generating N-terminal gasdermin fragments that form plasma membrane pores. Pyroptotic cells are characterized by cell swelling, membrane permeabilization, PI/7AAD entry, LDH release, and extracellular release of inflammatory mediators, including IL-1β, IL-18, ATP, HMGB1, DAMPs, and cytosolic contents. In cancer, pyroptosis has context-dependent effects: it may promote immunogenic tumor cell death, dendritic cell activation, CD8 + T cell and NK cell responses, and improved therapeutic response, but persistent inflammatory signaling may also support a pro-tumor microenvironment, angiogenesis, invasion, immunosuppression, and therapy resistance Created in BioRender. Kulbacka, J. (2026) https://BioRender.com/hi99tz7

Pyroptosis is a type of PCD originally described in response to bacterial infections but now recognized as a broader process involved in immunity, inflammation, and cancer biology as summarized in Fig. 2. It is important to note that pyroptosis has a dual role in cancer. With proper stimulation, it can promote tumor growth and progression, lead to tumor destruction, and suppress cancer. Recent studies reveal that pyroptosis-related genes and gasdermin family members are not only mechanistic effectors but also hold value as prognostic biomarkers and therapeutic targets across multiple cancers, including glioma, thyroid, breast, and ovarian cancer. Importantly, definitive evidence for pyroptosis requires genetic validation of caspase-1 or gasdermin dependency, as expression profiling alone is insufficient to establish its occurrence. According to studies, they can serve as prognostic indicators and potential therapeutic targets. Over time, there is an increasing number of pyroptosis markers, including activation of caspases-1, −4, −5, and − 11, GSDMD cleavage, and the release of interleukin-1β and interleukin-18. Due to the low molecular weight of the dyes in these staining methods, 7-aminoactinomycin (7-AAD), propidium iodide (PI), and ethidium bromide (EtBr), these methods can penetrate the pyroptotic cell membrane and stain those cells. Advanced computational methods, from functional assays to machine-learning–based models and risk indicators such as the PyroptosisScore, are increasingly being applied to predict survival outcomes and immunotherapy responses. Distinguishing pyroptosis from other lytic processes, such as necroptosis or ferroptosis, is critical, since pyroptosis typically features slower membrane rupture, cytoplasmic flattening, and vesicle-like pyroptotic bodies. Together, these insights underscore pyroptosis as a dynamic, clinically relevant cell death pathway whose regulation may be harnessed for both diagnostic and therapeutic innovation.

## Data Availability

Data sharing is not applicable to this article as no new data were created or analyzed in this study.
